# Readout and control of the spin-orbit states of two coupled acceptor atoms in a silicon transistor

**DOI:** 10.1126/sciadv.aat9199

**Published:** 2018-12-07

**Authors:** Joost van der Heijden, Takashi Kobayashi, Matthew G. House, Joe Salfi, Sylvain Barraud, Romain Laviéville, Michelle Y. Simmons, Sven Rogge

**Affiliations:** 1 School of Physics and Australian Centre of Excellence for Quantum Computation and Communication Technology, UNSW, Sydney, Australia.; 2 University of Grenoble Alpes and CEA, LETI, MINATEC, 38000 Grenoble, France.

## Abstract

Coupling spin qubits to electric fields is attractive to simplify qubit manipulation and couple qubits over long distances. Electron spins in silicon offer long lifetimes, but their weak spin-orbit interaction makes electrical coupling challenging. Hole spins bound to acceptor dopants, spin-orbit–coupled *J* = 3/2 systems similar to Si vacancies in SiC and single Co dopants, are an electrically active spin system in silicon. However, *J* = 3/2 systems are much less studied than *S* = 1/2 electrons, and spin readout has not yet been demonstrated for acceptors in silicon. Here, we study acceptor hole spin dynamics by dispersive readout of single-hole tunneling between two coupled acceptors in a nanowire transistor. We identify *m*_*J*_ = ±1/2 and *m*_*J*_ = ±3/2 levels, and we use a magnetic field to overcome the initial heavy-light hole splitting and to tune the *J* = 3/2 energy spectrum. We find regimes of spin-like (+3/2 to −3/2) and charge-like (±1/2 to ±3/2) relaxations, separated by a regime of enhanced relaxation induced by mixing of light and heavy holes. The demonstrated control over the energy level ordering and hybridization are new tools in the *J* = 3/2 system that are crucial to optimize single-atom spin lifetime and electrical coupling.

## Teaser

The controllable transition from charge to spin physics for a boron atom in silicon is an essential step to spin-orbit qubits.

## INTRODUCTION

Spins in silicon are promising qubits for future quantum information technologies with long spin coherence times in ^28^Si and the technological benefit of industry-compatible silicon fabrication processes. Great progress has been made on individual Si:P donor spin qubits including high-fidelity single-qubit logic gates and single-spin measurement (*1*, *2*). Prospects for building large quantum information systems in silicon could be greatly improved by coupling spin qubits to electric fields (*3*–*5*) to achieve rapid qubit manipulation and qubit coupling over large distances. However, electron spins in silicon experience weak intrinsic spin-orbit coupling, such that coupling these spin-based qubits to electric fields has, to date, required hybridizing the spin states with valley or charge eigenstates or the use of micrometer-scale magnets (*6*, *7*). These approaches increase the fabrication complexity and introduce extra decoherence channels. Hole spins in the valence band of silicon are an attractive alternative because they have a large intrinsic spin-orbit coupling, and electrical hole spin manipulation has recently been demonstrated in silicon (*8*).

The development of a single-atom qubit platform that leverages silicon’s long spin lifetimes in combination with electrical spin manipulation and long-distance coupling would be highly attractive. Single-acceptor dopant atoms offer strong spin-orbit coupling, making them interesting candidates for electrical manipulation (*9*–*15*). Unlike quantum dots, they offer identical confinement potentials, and similar to Si vacancies in SiC and single Co impurities (*16*–*19*), they offer the richness of a *J* = 3/2 manifold to engineer their properties (*9*–*12*). For example, it has been recently theoretically predicted that the features of these *J* = 3/2 manifolds in acceptors allow for electrically mediated long-distance coupling, together with long hole spin lifetimes (*11*, *12*), in addition to earlier predictions on phonon-mediated coupling (*9*). Yet to date, the properties of *J* = 3/2 systems are much less studied than their *S* = 1/2 counterparts. Hole quantum dots in silicon are not expected to have the same qubit engineering possibilities as the single-acceptor qubits due to the strongly broken degeneracy of the *J* = 3/2 manifold arising from the anisotropic confinement potential of the quantum dots. Moreover, their properties vary considerably from one report to another (*8*, *20*, *21*). To date, electrical readout of *J* = 3/2 systems has not been demonstrated, and the properties of acceptor-bound hole spins have received little attention in the context of quantum information applications.

Here, we demonstrate hole spin readout by detecting spin-dependent tunneling of a single hole between two coupled acceptor dopant atoms in a nanowire transistor fabricated using an industrial process. A dispersive coupling of the two-atom system to a resonator connected to the gate of the transistor realizes the spin readout, which allows us to probe spin relaxation in the closed two-atom system as a function of magnetic field (Zeeman energy ε_Z_). We also perform single-hole magnetotransport spectroscopy of the two-atom system and identify |*m*_*J*_| = 1/2 (light hole) and |*m*_*J*_| = 3/2 (heavy hole) levels within the *J* = 3/2 manifold and the characteristic splitting Δ_LH_ between them. Combining the singlet-triplet spin relaxation and magnetotransport spectroscopy, we identify regimes ε_Z_/Δ_LH_ < 1 and ε_Z_/Δ_LH_ > 1, where the two-atom dynamics is dominated by relaxation of spin-like (3/2 to −3/2) and charge-like (3/2 to 1/2) degrees of freedom, respectively. These two regimes are separated by a broad relaxation hotspot region of enhanced relaxation induced by hybridization of the |*m*_*J*_| = 3/2 and |*m*_*J*_| = 1/2 degrees of freedom. In particular, the demonstrated control over the interplay between magnetic (ε_Z_) and electric splitting (Δ_LH_), together with the hybridization of 3/2 and 1/2 levels, are the capabilities necessary to optimize spin relaxation, decoherence, and qubit coupling to electric fields. In this way, the benefits of single-atom qubits, such as reproducibly long lifetimes, could potentially be combined with electrically mediated long-distance qubit coupling.

## RESULTS

### Identification of individual acceptors

**Fig. 1:**
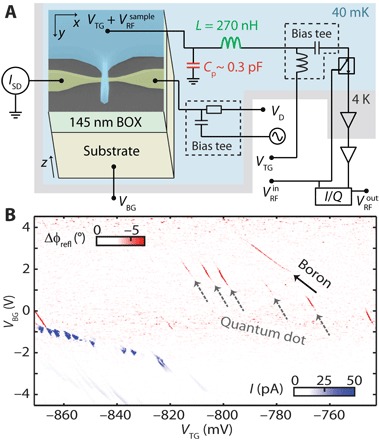
Detection of a single acceptor atom. (**A**) Scanning electron microscopy image of the used tri-gate transistor in a schematic diagram of the experimental setup for transport and RF gate reflectometry measurements. The magnetic field in this experiment is applied along the *y* direction (along the channel). (**B**) Change in reflected phase from the LC circuit (red) compared to the measured source-drain current (blue), both at 1 mV *V*_D_. The signals of a boron atom (black arrow) and an unintentional quantum dot (gray arrows) are identified. BOX, buried oxide.

We use radio frequency (RF) gate reflectometry (*22*, *23*) to detect the charging of localized states in the nanowire transistor, which has previously been used to detect single electrons bound to donors in Si (*24*), as well as their intersite transitions (*25*). This charging translates to a shift of the phase, Δϕ_refl_ or amplitude, Δ*A*_refl_, of an RF excitation reflected from an LC circuit connected to the top gate, when driven near the resonance frequency with *V*_RF_. These shifts are a result of the change in admittance of the LC circuit, induced by spin-selective tunneling (*26*) of holes to and from localized sites. In the measured map of top gate voltage (*V*_TG_) and back gate voltage (*V*_BG_) shown in Fig. 1B, charging lines appear below the turn on voltage of the nanowire transistor channel (see fig. S1A) because of tunneling back and forth between a localized site and one of the reservoirs. These lines often only appear in small back gate voltage ranges because of the back gate dependence of both the tunnel rates and of the total capacitance of the sensing gate. The states of neutral and positively charged boron atoms are anticipated to contribute to charge transitions furthest below the turn on voltage, as these states are found within the bandgap of silicon. Series of localized states with identical slopes (gray arrows in Fig. 1B) reflect charging events on the same site, likely to be an unintentional quantum dot at the Si/SiO_2_ interface (*27*), while boron atoms bind no more than two holes (*28*). Another localized site, comparatively less strongly capacitively coupled to the top gate and unaccompanied by lines of equal slope (black arrow in Fig. 1B), indicates the charging of the first hole onto a single-boron atom. We estimate the location of this atom by comparing the capacitive couplings to all four transistor electrodes (see Materials and Methods) and find a position close to the drain lead, inconsistent with a gate-defined quantum dot below the top gate.

**Fig. 2:**
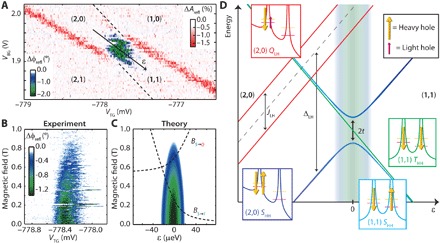
Two-hole system. (**A**) Reflected amplitude and phase response showing a break in the acceptor-lead charging line (red) caused by the charging of a nearby acceptor atom (dashed lines). (**B** and **C**) Measured and calculated magnetic field dependence of the interacceptor tunneling phase response. The magnetic fields yielding the singlet-triplet crossing *B*_*S*→*T*_ and singlet-quadruplet crossing *B*_*S*→*Q*_ are plotted in (C) as a function of detuning by dashed curves. (**D**) Schematic overview of the relevant two-hole states. The (1,1) and (2,0) *S*_HH_ states (blue), (1,1) *T*_HH_ states (green), and (2,0) *Q*_LH_ states (red) are shown. While this subset of two-hole states is sufficient to describe our experiments, a full description of the spin-orbit states can be found in section S3. Insets show a depiction of the potential landscape and possible spins involved in these states.

A sudden shift in the charging line of this acceptor, shown in Fig. 2A, is the signature of Coulombic interactions with a nearby localized site upon a change in its charge occupation. As we will subsequently argue, the directly probed excited state spectrum of this nearby site is inconsistent with a quantum dot and is expected for a boron atom binding two holes. The negative Δϕ_refl_ found within the break of the line indicates the tunneling of holes between the two atoms. A detailed study of the reflected signal in the frequency domain shows that the acceptor-lead tunnel rate is comparable to the resonance frequency (583 MHz) of the LC circuit. In contrast, we find the interacceptor tunnel rate to be much faster than this resonance frequency. We do not observe the tunneling of holes from either lead to the second acceptor atom, likely because of tunnel rates much slower than the resonance frequency. For a complete analysis of all the involved tunnel rates, see section S1 and figs. S1B and S2.

### Dispersively detected Pauli spin blockade in the two-hole system

The magnetic field dependence of the interacceptor signal, shown in Fig. 2B, provides information on the charge occupation of the two-acceptor system. We observe the signature of Pauli spin blockade via the suppression of intersite tunneling with increasing magnetic field (*29*, *30*). This occurs because spins in the triplet state, of which the population increases because of its lowering in energy in the applied magnetic field, cannot tunnel to a singlet state because of the Pauli exclusion principle (see Materials and Methods). This observation of spin-selective tunneling shows that there is an even number of holes bound to the two acceptor atoms. Since acceptors can bind up to two holes (*28*), we conclude that the observed interacceptor tunneling is a (1,1)↔(2,0) transition.

The two-hole states of the interacting acceptors (see figs. S4 and S5) reflect the low-energy *J* = 3/2 manifold that includes both ±3/2 and ±1/2 states, separated by 20 meV from any additional excited states. In the absence of magnetic fields, an electric potential can split the fourfold degeneracy into two doublets (*14*, *31*, *32*), while the presence of magnetic fields will split these doublets via the Zeeman interaction. Figure 2D shows the total energy of the two-hole system at zero magnetic field as a function of the energy level detuning ε between the atoms, assuming heavy-hole ground states that are consistent with our experiments. To interpret our measurements, it is sufficient to only consider the lowest spin manifold for the (1,1) configuration (right side of Fig. 2D), where one heavy hole resides on each acceptor, forming a singlet state, *S*_HH_, and three triplet states, *T*_HH_^−^, *T*_HH_^0^, and *T*_HH_^+^. For the (2,0) configuration (left side of Fig. 2D), the doubly occupied acceptor A^+^ state, the exchange energy between holes with the same angular momentum (*J*_HH_ and *J*_LL_) or different angular momenta (*J*_LH_ = *J*_HL_) obey *J*_HH_, *J*_LL_ > Δ_LH_ > *J*_LH_ in our experiments. In analogy to the two-hole states of neutral group II acceptors (*33*) and of two closely spaced boron atoms (*15*), the lowest energy manifold in the (2,0) configuration consists of six states, split by Δ_LH_ and *J*_LH_. The ground state is the heavy hole singlet, *S*_HH_. Four states consisting of one heavy hole and one light hole, *Q*_LH_^*i*^, with *i* denoting the angular momentum, are approximately Δ_LH_ higher in energy and split by *J*_LH_ into singlet and triplet states, as shown in Fig. 2D. The sixth state is the light hole singlet state, *S*_LL_, approximately another Δ_LH_ higher in energy. For most of the presented analysis, it is sufficient to only consider the *S*_HH_, *T*_HH_^−^, and *Q*_LH_^2−^ states, which, for simplicity, we will from now on refer to as the *S*, *T*, and *Q* states.

At zero magnetic field, the tunnel coupling *t* between the (1,1) *S* and (2,0) *S* states gives rise to the interacceptor reflectometry signal, with *t* measured by a microwave excitation experiment to be 4.3 ± 0.3 GHz (see section S1 and fig. S3) (*34*). At a finite magnetic field, which we label *B*_*S*→*T*_, the (1,1) *T* state becomes the ground state at the zero detuning point. Using the tunnel coupling *t* and the thermal occupation of the two-hole states (see Materials and Methods), we simulate this magnetic field dependence of the reflectometry signal (*35*), as shown in Fig. 2C. From the simulation, we estimate *B*_*S*→*T*_ at 0.25 ± 0.05 T, which provides an effective Landé *g* factor (g* = *gm*_*J*_) of the *T* state of 1.2 ± 0.3, using the Zeeman energy of g*μ_B_B, where μ_B_ is the Bohr magneton. In this low–magnetic field regime, we find that g* is suppressed compared to an acceptor state with cubic site symmetry, for which Δ*m*_*J*_ = 3 and *g* ≈ 1 (g* ≈ 3) are expected. For an acceptor atom, this suppression of g* can occur when the magnetic field splitting is smaller than the heavy-light hole splitting set by an electric potential that could originate from local strain, an electric field, or a nearby interface (fig. S4B).

### Relaxation and hybridization of ±3/2 and ±1/2 states

**Fig. 3:**
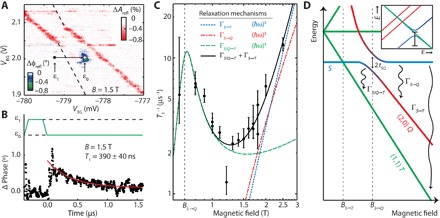
Spin relaxation mechanisms of the two-hole system. (**A**) Time-averaged RF reflectometry amplitude (red) and phase (blue) measurement with continuous pulses applied to the drain, at a magnetic field of 1.5 T. The interacceptor tunneling is displaced from the break of the acceptor-lead tunneling signal by the Zeeman energy of the *T* ground state. (**B**) Relaxation time extracted from the time-dependent phase signal for a pulse between the points ε_0_ and ε_1_. (**C**) Magnetic field dependence of the relaxation rate *T*_1_^−1^. (**D**) Schematic view of the relevant relaxation rates in this system at the zero detuning point (see inset). The data in (C) are fit to a combination of the Γ_*S*/*Q*→*T*_ rate (green line) and Γ_*S*→*T*_ rate (blue line), resulting in the black line. This is compared to a fit using the Γ_*S*→*Q*_ rate (red line) instead of the Γ_*S*→*T*_ rate.

We investigated spin relaxation of the two-hole system by time-dependent gate reflectometry using pulsed gate voltages. Specifically, we generate a nonequilibrium singlet population by pulsing the system via the (2,1) region into the (2,0) state and monitoring the intersite tunneling at the (1,1)↔(2,0) degeneracy point. The asymmetry in tunnel rates to both acceptors (see section S1) ensures that the charge transition via the (2,0) state is much more likely than the direct transition from the (2,1) state to the (1,1) state in this pulse scheme. The nonequilibrium singlet tunneling signal, indicated by the white circle in Fig. 3A, is obtained by pulsing 0.8 mV on the drain electrode to the readout point ε_0_ for 160 ns, after spending 40 ns at ε_1_, in a magnetic field of 1.5 T. The transient decay of this phase response shown in Fig. 3B reflects the singlet probability decay by relaxation at the degeneracy point (*36*), averaged over 10^6^ sequences, for the modified pulse timing shown in the inset of Fig. 3B and the same detunings ε_0_ and ε_1_ as in Fig. 3A.

By fitting the measured singlet decay for different magnetic fields, we obtain the relaxation rates in Fig. 3C. Above 1.5 T, T_1_^−1^ increases with magnetic field in agreement with spin-lattice relaxation (*9*, *37*), but below 1.5 T, T_1_^−1^ reaches a maximum around ~0.8 T, which is not expected for spin-lattice relaxation. We also rule out nuclear spin–induced hole spin relaxation as this would occur at smaller magnetic fields. Rather, these measurements resemble the relaxation observed at degeneracy points for spin and orbital degrees of freedom (*38*, *39*). A schematic of the magnetic field dependence of two-hole states (Fig. 3D) identifies degeneracy points *B*_*S*→*T*_, where the (1,1) *T* state falls below the singlet, and *B*_*S*→*Q*_, where the (2,0) *Q* state falls below the singlet. We fit the data in Fig. 3C to a model that takes into account various relaxation mechanisms. These include the spin-lattice relaxation Γ_*S*→*T*_ from the *S* state to the *T* state, which requires a heavy hole spin flip (Δ*m*_*J*_ = 3 relaxation), and the relaxation Γ_*S*→*Q*_ from the *S* state to the *Q* state, which requires a flip from a light hole to a heavy hole (Δ*m*_*J*_ = 2 relaxation). Both of these spin-lattice relaxation rates have a monotonic power law dependence on magnetic field, as described by

$$\Gamma_{S\to T[Q]}=A_{ST[Q]}{\left({\left(B-B_{S\to T[Q]}\right)}^{2}+{\left(2t_{ST[Q]}\right)}^{2}\right)}^{\gamma_{ST[Q]}/2}$$ (1)

with *A*_*ST*[*Q*]_ being the relaxation amplitude for the *S*→*T*[*Q*] process and *t*_*ST*[*Q*]_ being the coupling of the *S* state and the (1,1) *T* [(2,0) *Q*] state. A γ_*SQ*_ of 3 characterizes the Δ*m*_*J*_ = 2 (light to heavy hole) relaxation because it has a strong charge-like character and is therefore directly allowed by phonons. The Δ*m*_*J*_ = 3 relaxation is characterized by γ_ST_ = 5 and should be weaker than the Δ*m*_*J*_ = 2 transition, since it involves a less direct heavy hole spin flip (*9*, *11*).

A light-heavy hole tunneling-induced hybridization between the *S* and *Q* states causes the anticrossing at *B*_*S*→*Q*_ (Fig. 3D). Here, we expect a spin relaxation hotspot (*38*, *40*, *41*), since the finite light hole component opens a light-to-heavy hole relaxation path to the (1,1) *T* state with relaxation rate Γ_*S*/*Q*→*T*_, which is expected to be much faster than the heavy-to-heavy hole relaxation process Γ_*S*→*T*_ (*9*, *11*). This relaxation strongly depends on the light hole component in the hybridized states (see Materials and Methods), and we obtain

$$\Gamma_{S/Q\to T}=A_{QT}\frac{{2t_{SQ}}^{2}{\left(B-B_{S\to T}\right)}^{3}}{{\left(B-B_{S\to Q}\right)}^{2}+{\left(2t_{SQ}\right)}^{2}}$$ (2)

with *A*_*QT*_ being the relaxation amplitude between the *Q* and *T* states.

The sum of Γ_*S*→*T*_ and Γ_*S*/*Q*→*T*_ gives a satisfactory least squares fit to the magnetic field dependence of *T*_1_^−1^, as shown by a solid black line in Fig. 3C. Our fit yields *B*_*S*→*Q*_ = 0.70 ± 0.02 T at the anticrossing point and an asymmetry *A*_*QT*_/*A*_ST_ ≈ 1.2 × 10^3^, although we could not determine the exponent of the spin relaxation mechanism above 1.5 T, as shown in fig. S6 (see also section S4). The large asymmetry *A*_*QT*_/*A*_*ST*_ reflects the faster Δ*m*_*J*_ = 2 relaxation (light to heavy hole) compared to Δ*m*_*J*_ = 3 relaxation (heavy hole up to heavy hole down), as expected because the Δ*m*_*J*_ = 2 relaxation is mediated directly by phonons.

The spectral width of the hotspot in Fig. 3C reflects the direct tunnel coupling–induced hybridization between the *Q* state, which contains one heavy hole and one light hole, and the *S* state of two heavy holes. Our fit of the *T*_1_ lineshape yields a tunnel coupling *t*_*SQ*_ = 0.04 ± 0.015 T, which converts to an energy between 3.5 μeV (840 MHz) and 7 μeV (1.6 GHz), assuming g* between 1.5 and 3.0. Since the singlet is actually a superposition of (2,0) and (1,1) charge configurations, *t*_*SQ*_ includes contributions involving tunneling to the other atom [for the (1,1) singlet] and no tunneling to the other atom [for the (2,0) singlet]. Given that the intersite tunnel coupling of the heavy holes is 4.3 GHz (fig. S3), and heavy–to–light hole intersite tunneling is expected to be a factor of ~10 weaker (~430 MHz) (*15*), we expect that the bulk of the tunneling between the light and heavy holes is an on-site hybridization that occurs on the same atom.

This on-site hybridization of |*m*_*J*_| = 1/2 and 3/2 states is a key property of quantum bits in *J* = 3/2 systems. Increasing the degree of hybridization can be used to increase the splitting Δ to enhance qubit relaxation times (*9*–*12*) or to mix the |*m*_*J*_| = 1/2 with the |*m*_*J*_| = 3/2 states to obtain fast electric drive, together with charge-noise insensitivity at sweet spots (*10*, *11*). Assuming that the mixing is caused by the lowest-order electric field coupling, we find an electric field component *E* = *t*_SQ_/*p* ~ 0.17 MV/m to 0.34 MV/m causing the anticrossing at the degeneracy point, where *p* ≈ 0.26 D is the *T*_d_ symmetry light–to–heavy hole dipole coupling (*42*, *43*). For large gate electric fields (20 MV/m), which can be tolerated near interfaces, these couplings extrapolate to ~100 GHz light–to–heavy hole mixings. As required to realize the theoretically predicted sweet spots (*10*, *11*), the mixing at the high gate field exceeds typical qubit Larmor frequencies. Notably, the larger gate fields are also required to obtain a very strong coupling of the qubit state to electric fields via a Rashba-like interaction (*10*, *11*).

### Tuning the two-hole spectrum

**Fig. 4:**
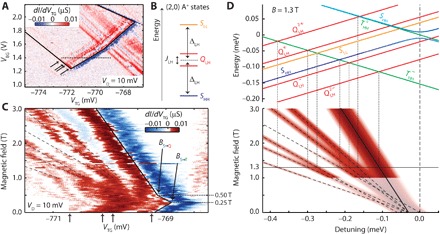
Excited states of the two-hole system measured by transport spectroscopy. (**A**) Transconductance *dI*/*dV*_TG_ as a function of the top and back gate voltages at 10 mV *V*_D_. The bias triangle, marked by solid black lines, shows three excited states in addition to the ground state as indicated by arrows. (**B**) Level configuration of the A^+^ state, corresponding to the found excited states. (**C**) Magnetic field dependence of the transconductance at a cut through the bias triangle at 1.4 V *V*_BG_, as indicated by the dotted line in (A). At the bottom, arrows indicate the excited state spectrum as also shown in (A). Two changes in the slope of the ground state at 0.25 and 0.5 T are the result of the *B*_*S*→*T*_ and *B*_*S*→*Q*_ crossings. (**D**) Model of the transition energies in the high–magnetic field limit. Top: Overview of the (1,1)→(2,0) transitions at a magnetic field of 1.3 T. Bottom: Simulated magnetic field behavior of the transition energy spectrum, using the *g* factors and Δ_LH_ extracted from the transition energies in (C) and using the same line broadening as found in this experiment.

To further understand the coupled acceptor system, we perform excited state spectroscopy by single-hole tunneling transport (*13*, *20*, *21*). In Fig. 4A, the transconductance signal as a function of *V*_TG_ and *V*_BG_ at zero magnetic field shows a bias triangle (contour marked by a solid line), demonstrating sequential hole tunneling via both acceptors in series. We attribute lines parallel to the triangle baseline (arrows in Fig. 4A) to transitions from the (1,1) ground state to the (2,0) excited states (see section S5 and figs. S4 and S5). The observed (2,0) spectrum matches the expected energy spectrum of a doubly charged acceptor state, as shown in Fig. 4B. This supports our identification of the second confinement site as an acceptor atom, further validated by the incompatibility of our measurement with the regularly spaced excited state spectrum expected for quantum dots. The (2,0) Δ_LH_ and *J*_LH_ are extracted as 110 ± 10 μeV and 36 ± 5 μeV, respectively. The exchange splitting *J*_LH_ is not to be confused with the tunnel coupling between light and heavy hole states at the magnetic field–induced degeneracy. Rather, it describes the energy lowering of two-hole states on a single atom due to hybridization induced by Coulomb interactions (*33*). Nevertheless, *J*_LH_ reflects a tunneling process between light and heavy holes, so it is not unexpected that it is of the order of *t*_*SQ*_.

The splitting Δ_LH_ between the *m*_*J*_ = 3/2 and *m*_*J*_ =1/2 states at zero magnetic field is a key parameter for qubits in *J* = 3/2 systems, since its interplay with the Zeeman interaction ε_Z_ determines the qubit properties (*9*, *42*). The two-level system formed by the lowest-energy states is charge-like when ε_Z_ > |Δ_LH_| and spin-like when ε_Z_ < |Δ_LH_|. For an acceptor in silicon, a nonzero Δ_LH_ can only reflect a lowered symmetry of the acceptor state due to an electric potential that lacks the cubic symmetry of the silicon lattice (*31*, *42*). The perturbation of this electric potential causes mixing and splitting of the *J* = 3/2 states independently of the Zeeman interaction, resulting in a host of distinct properties (*43*) that are absent for conventional *S* = 1/2 electron spins. Especially, when this perturbation dominates the Zeeman interaction (ε_Z_ < Δ_LH_) and is applied along a different direction from the magnetic field, it results in a reduced g* (*43*).

To investigate the crossover from ε_Z_ < |Δ_LH_| to ε_Z_ > |Δ_LH_|, we compare the transition energy spectrum including high magnetic field data with the spin blockade data at low magnetic fields. We measured the excited state data as a function of magnetic field, as shown in Fig. 4C, on a cut through the bias triangle at *V*_BG_ = 1.4 V (dotted line in Fig. 4A). The baseline of the triangle, indicated with a solid line in Fig. 4C, changes its slope twice. The first change around 0.25 T indicates the singlet-triplet crossing at *B*_*S*→*T*_, in agreement with the observation of spin blockade in Fig. 2B. The second change around 0.50 T indicates the change of the (2,0) ground state from *S* to *Q*, corresponding to the same anticrossing that generates the relaxation hotspot in Fig. 3B but here occurring in the negative detuning region and therefore at a lower magnetic field.

In the high–magnetic field regime (>1 T), we observe clear resonance lines outnumbering those at zero magnetic field, indicating spin-split excited states. In this regime, the Zeeman energy dominates over Δ_LH_, and therefore, the angular momenta of the two-hole states align with the external magnetic field. Ignoring any mixing terms other than the mixing of the *S* state, the magnetic field dependence of the transition energies can be easily described by using *m*_*J*_ = ±3/2 for heavy holes and *m*_*J*_ = ±1/2 for light holes to calculate the Zeeman shifts, as shown in Fig. 4D. Using this model, we extract the *g* factors and Δ_LH_ by fitting the slopes and position of the transition lines in Fig. 4C, respectively. For the doubly occupied acceptor, we find *g*_3/2_ of 0.85 and *g*_1/2_ of 1.07 and Δ_LH_ of 30 μeV. For the singly occupied acceptor, only the heavy hole states play a role in the transitions for which we extract *g*_3/2_ of 1.05. Note that, here, we use *m*_*J*_ to determine the *g* factor instead of the effective *g* factor g*.

A comparison of the low–magnetic field (spin blockade) and high–magnetic field (transport spectroscopy) measurements demonstrates the changes in the *J* = 3/2 system between the ε_Z_ < |Δ_LH_| and ε_Z_ > |Δ_LH_| regimes. The *g* factors found in the high–magnetic field regime are close to those found for bulk boron acceptors (*44*, *45*), contrasting the smaller effective *g* factor found at low magnetic fields in the spin blockade experiment in Fig. 2B, when the acceptor system is dominated by the perturbation of an electric potential. Furthermore, the Δ_LH_ of the doubly occupied acceptor is about four times smaller in the high–magnetic field regime than at zero magnetic field. A further indication of the crossover between different regimes of the *J* = 3/2 system is the appearance of transport signal attributed to the (1,1) *T* to (2,0) *S* transition when ε_Z_ < |Δ_LH_|. This transition is prohibited by the Pauli principle when the total angular momenta are aligned with the external magnetic field, resulting in the absence of a corresponding transport feature at magnetic fields higher than 2 T. At small magnetic fields, on the other hand, the mixing of the *J* = 3/2 states allows this transition, and thus, the corresponding transport feature appears. The crossover between ε_Z_ < |Δ_LH_| and ε_Z_ > |Δ_LH_| reflects a controlled transition between the regimes dictated by spin physics and charge physics, which is a unique feature of the *J* = 3/2 systems.

## DISCUSSION

We have demonstrated the readout of the spin state of two coupled atoms using a reflected RF signal from a resonator connected to the gate of a nanowire transistor and the Pauli exclusion principle (*46*). Given that the readout scheme requires a tank circuit on the gate electrode and avoids the use of a single-electron transistor, it could be exploited to simplify the readout in a scalable quantum computing architecture. Using this spin readout scheme, we have investigated the lifetime of spin excitations at the charge degeneracy point of the two atoms and found a magnetic field–induced anticrossing caused by hybridization between |*m*_*J*_| = 3/2 and |*m*_*J*_| = 1/2 spin degrees of freedom. Combined with transport spectroscopy data, we also found that it is possible to tune between regimes defined by the heavy-light hole splitting and defined by the Zeeman energy. This allows the change between a spin physics– and a charge physics–dominated system, thereby controlling the *g* factor and heavy-light hole splitting of the *J* = 3/2 system, which has no analog in a *S* = 1/2 system. In the future, larger splittings Δ_LH_ induced by confinement or strain can be used to enhance qubit lifetime, and higher gate electric fields can be used to enhance qubit coupling to electric fields.

## MATERIALS AND METHODS

### Experimental design

The silicon nanowire transistor used in this work was fabricated using a standard complementary metal-oxide semiconductor technology, similar to previously reported devices (*21*, *24*). The channel, defined by the top gate, is 11 nm high, 42 nm wide, and 54 nm long. On both sides of this gate, 25-nm-long Si_3_N_4_ spacers form barriers to the highly doped source and drain regions. With a background boron doping of 5 × 10^17^ cm^−3^ in the silicon channel, about 10 boron atoms are expected under the gate and about 10 are expected under the spacers.

In a dilution refrigerator at a base temperature of 40 mK, hole transport and RF gate reflectometry measurements give complementary insights into the static and dynamic behavior of the two-hole system of interest. The hole temperature was measured from quantum dot transport at ~ 300 mK. The noise floor of the transport measurement (~100 fA) allows us to detect currents with a total tunnel rate down to 500 kHz. A surface mount 270-nH inductor, attached to the top gate, forms a tank circuit together with a parasitic capacitance of ~0.3 pF. Tunnel events in the transistor with a tunnel rate higher than or equal to the resonance frequency of 583 MHz result in a change of the reflected amplitude and/or phase of this resonator. Furthermore, low-intensity light was directed to the silicon chip through an optical fiber to enable the use of the lower doped substrate as a back gate.

### Location of the acceptors

To estimate the location of the two atoms, we extracted the relative capacitive coupling to the different electrodes. The hole temperature of ~300 mK gives *k*_B_*T* >> *ℏ*Γ for the acceptor-lead tunnel rate, thereby justifying a thermally broadened fit (*25*). The reflectometry linewidth shows a relative coupling to the top gate (α_TG_) and drain (α_D_) of 0.50 ± 0.01 eV/V and 0.41 ± 0.02 eV/V, respectively, thereby placing this atom roughly halfway between these two electrodes, under the Si_3_N_4_ spacer. This relatively weak coupling to the top gate supports our identification of the acceptor atom, as gate-induced quantum dots are known to have gate couplings above ~0.7 eV/V (*24*, *27*). The second boron atom has to be located closer to the center of the channel, as the tunneling to both electrodes is too slow to be detected.

### Pauli spin blockade in RF gate reflectometry

The shift in phase response of the reflected signal, as shown in Fig. 2B, is ascribed to the addition of a quantum capacitance (*C*_Q_) to the LC circuit (*22*–*25*) when holes can tunnel between the two atoms, given by

$$C_{Q}=\frac{1}{2}\Delta \alpha_{\text{TG}}^{2}q^{2}{4t}^{2}{\left(\varepsilon^{2}+2t^{2}\right)}^{-3/2}$$ (3)

where Δα_TG_ is the difference in capacitive coupling from the top gate to each acceptor (found to be 0.14 eV/V in the transport measurements), *q* is the electron charge, ε is the energy detuning between the two acceptors, and *t* is the tunnel coupling between the (1,1) and (2,0) *S* states. All other tunnel couplings between the (1,1) and (2,0) states are considered to be much smaller and therefore not contributing to a change in the detected reflected signal. The total phase shift (Δϕ) was determined by the difference in occupation, given by the Boltzmann distribution, of the lower and upper branches of the heavy hole singlet state (*35*)

$$\Delta \phi \sim C_{Q}\frac{e^{\left(-E_{S^{-}}/kT\right)}-e^{\left(-E_{S^{+}}/kT\right)}}{\sum e^{\left(-E_{i}/kT\right)}}$$ (4)

where *E*_S−_ and *E*_S+_ are the energies of the bonding and antibonding singlet states, respectively, and *E*_i_ describes the energies of all the involved states. Using Eqs. 3 and 4, the magnetic field dependence of the phase shift caused by the interacceptor tunneling was calculated, as shown in Fig. 2C. We note that, although the crossing of the *Q* state and the *S* state has some influence on the Boltzmann distribution of the *S* states, this influence is not distinguishable in the measurement. However, for completeness, we displayed the *B*_*S*→*Q*_ crossing point in Fig. 2C, as found in the relaxation hotspot measurement, together with an estimated *g* factor of the *Q* state.

### Relaxation hotspot

The observed relaxation hotspot is explained by the mixing of the bonding *S* state and the (2,0) *Q* state, which allows a light–to–heavy hole relaxation to the (1,1) *T* state. This relaxation process is expected to have a (*ℏ*ω)^3^ dependence and furthermore depends linearly on the light hole character of the mixed state. Here, we show the calculation of the light hole character of the probed state. Around the anticrossing, we can write the energies of the lower (−) and upper (+) states as

$$E_{\pm }=\frac{\Delta E}{2}\pm \frac{1}{2}\sqrt{{\left(\Delta E\right)}^{2}+{\left(2t_{SQ}\right)}^{2}}$$ (5)

where Δ*E* is the energy splitting between the states in the absence of mixing (*E*_*Q*_ − *E*_*S*_), and *E*_*S*_ is taken as the reference energy (*E* = 0). We defined the amount of light hole character of each state as the chance to measure it as the *Q* state in the basis of *Q* and *S*, written as ξ_±_ and given by

$$\xi_{\pm }=\frac{1}{2}\frac{\Delta E}{2\sqrt{{\left(\Delta E\right)}^{2}+{\left(2t_{SQ}\right)}^{2}}}$$ (6)

The relaxation measurement was performed at zero detuning, which is the original position of *E*_*S*_ in the absence of mixing. The state probed here can be written as a combination of the positive (+) and negative (−) states, depending on their relative energy difference with the measurement point |*E*_±_|/(|*E*_−_| + |*E*_+_|). We note that $|E_{-}|\xi_{-}=|E_{+}|\xi_{+}={t_{SQ}}^{2}/\sqrt{{\left(\Delta E\right)}^{2}+{\left(2t_{SQ}\right)}^{2}}$ and also that (|*E*_−_| + |*E*_+_|) is equal to $\sqrt{{\left(\Delta E\right)}^{2}+{\left(2t_{SQ}\right)}^{2}}$, which gives the total light hole character at the zero energy point as

$$\xi_{E=0}=\frac{|E_{-}|\xi_{-}+|E_{+}|\xi_{+}}{\left|E_{-}|+|E_{+}\right|}=\frac{{2t_{SQ}}^{2}}{{\left(\Delta E\right)}^{2}+{\left(2t_{SQ}\right)}^{2}}$$ (7)

as used in Eq. 2.

## Supplementary Material

http://advances.sciencemag.org/cgi/content/full/4/12/eaat9199/DC1

## SUPPLEMENTARY MATERIALS

Supplementary material for this article is available at http://advances.sciencemag.org/cgi/content/full/4/12/eaat9199/DC1 Section S1. Estimation of tunnel rates Section S2. Acceptor-based single-atom qubits Section S3. Two-hole states Section S4. Relaxation mechanisms Section S5. (1,1)→(2,0) transition energies Fig. S1. Transport spectroscopy images. Fig. S2. Frequency domain analysis of the reflectometry signal. Fig. S3. Interacceptor continuous-wave microwave excitation experiment. Fig. S4. Examples of the Zeeman effect on *A*^0^ states. Fig. S5. Examples of the Zeeman effect on two-hole states. Fig. S6. Relaxation rate models. References (*47*–*51*)

## References

[1] Pla J. J., Tan K. Y., Dehollain J. P., Kim W. H., Morton J. J. L., Jamieson D. N., Dzurak A. S., Morello A., A single-atom electron spin qubit in silicon. Nature 489, 541–545 (2012). 22992519 10.1038/nature11449

[2] Muhonen J. T., Dehollain J. P., Laucht A., Hudson F. E., Kalra R., Sekiguchi T., Itoh K. M., Jamieson D. N., McCallum J. C., Dzurak A. S., Morello A., Storing quantum information for 30 seconds in a nanoelectronic device. Nat. Nanotechnol. 9, 986–991 (2014). 25305745 10.1038/nnano.2014.211

[3] Nowack K. C., Koppens F. H. L., Nazarov Yu. V., Vandersypen L. M. K., Coherent control of a single electron spin with electric fields. Science 318, 1430–1433 (2007). 17975030 10.1126/science.1148092

[4] Pioro-Ladrière M., Obata T., Tokura Y., Shin Y.-S., Kubo T., Yoshida K., Taniyama T., Tarucha S., Electrically driven single-electron spin resonance in a slanting Zeeman field. Nat. Phys. 4, 776–779 (2008).

[5] Medford J., Beil J., Taylor J. M., Bartlett S. D., Doherty A. C., Rashba E. I., DiVincenzo D. P., Lu H., Gossard A. C., Marcus C. M., Self-consistent measurement and state tomography of an exchange-only spin qubit. Nat. Nanotechnol. 8, 654–659 (2013). 23995458 10.1038/nnano.2013.168

[6] Takeda K., Kamioka J., Otsuka T., Yoneda J., Nakajima T., Delbecq M. R., Amaha S., Allison G., Kodera T., Oda S., Tarucha S., A fault-tolerant addressable spin qubit in a natural silicon quantum dot. Sci. Adv. 2, e1600694 (2016). 27536725 10.1126/sciadv.1600694 PMC4982751

[7] Kim D., Shi Z., Simmons C. B., Ward D. R., Prance J. R., Koh T. S., Gamble J. K., Savage D. E., Lagally M. G., Friesen M., Coppersmith S. N., Eriksson M. A., Quantum control and process tomography of a semiconductor quantum dot hybrid qubit. Nature 511, 70–74 (2014). 24990747 10.1038/nature13407

[8] Maurand R., Jehl X., Kotekar-Patil D., Corna A., Bohuslavskyi H., Laviéville R., Hutin L., Barraud S., Vinet M., Sanquer M., De Franceschi S., A CMOS silicon spin qubit. Nat. Commun. 7, 13575 (2016). 27882926 10.1038/ncomms13575 PMC5123048

[9] Ruskov R., Tahan C., On-chip cavity quantum phonodynamics with an acceptor qubit in silicon. Phys. Rev. B 88, 064308 (2013).

[10] Salfi J., Tong M., Rogge S., Culcer D., Quantum computing with acceptor spins in silicon. Nanotechnology 27, 244001 (2016). 27171901 10.1088/0957-4484/27/24/244001

[11] Salfi J., Mol J. A., Culcer D., Rogge S., Charge insensitive single-atom spin-orbit qubit in silicon. Phys. Rev. Lett. 116, 246801 (2016). 27367400 10.1103/PhysRevLett.116.246801

[12] Abadillo-Uriel J. C., Calderón M. J., Interface effects on acceptor qubits in silicon and germanium. Nanotechnology 27, 024003 (2016). 26618443 10.1088/0957-4484/27/2/024003

[13] van der Heijden J., Salfi J., Mol J. A., Verduijn J., Tettamanzi G. C., Hamilton A. R., Collaert N., Rogge S., Probing the spin states of a single acceptor atom. Nano Lett. 14, 1492–1496 (2014). 24571637 10.1021/nl4047015

[14] Mol J. A., Salfi J., Rahman R., Hsueh Y., Miwa J. A., Klimeck G., Simmons M. Y., Rogge S., Interface-induced heavy-hole/light-hole splitting of acceptors in silicon. Appl. Phys. Lett. 106, 203110 (2015).

[15] Salfi J., Mol J. A., Rahman R., Klimeck G., Simmons M. Y., Hollenberg L. C. L., Rogge S., Quantum simulation of the Hubbard model with dopant atoms in silicon. Nat. Commun. 7, 11342 (2016). 27094205 10.1038/ncomms11342 PMC4842981

[16] Kraus H., Soltamov V. A., Riedel D., Väth S., Fuchs F., Sperlich A., Baranov P. G., Dyakonov V., Astakhov G. V., Room-temperature quantum microwave emitters based on spin defects in silicon carbide. Nat. Phys. 10, 157–162 (2014).

[17] Widmann M., Lee S.-Y., Rendler T., Son N. T., Fedder H., Paik S., Yang L.-P., Zhao N., Yang S., Booker I., Denisenko A, Jamali M., Momenzadeh S. A., Gerhardt I., Ohshima T., Gali A., Janzén E., Wrachtrup J., Coherent control of single spins in silicon carbide at room temperature. Nat. Mater. 14, 164–168 (2015). 25437256 10.1038/nmat4145

[18] Otte A. F., Ternes M., von Bergmann K., Loth S., Brune H., Lutz C. P., Hirjibehedin C. F., Heinrich A. J., The role of magnetic anisotropy in the Kondo effect. Nat. Phys. 4, 847–850 (2008).

[19] Toskovic R., van den Berg R., Spinelli A., Eliens I. S., van den Toorn B., Bryant B., Caux J.-S., Otte A. F., Atomic spin-chain realization of a model for quantum criticality. Nat. Phys. 12, 656–660 (2016).

[20] Li R., Hudson F. E., Dzurak A. S., Hamilton A. R., Pauli spin blockade of heavy holes in a silicon double quantum dot. Nano Lett. 15, 7314–7318 (2015). 26434407 10.1021/acs.nanolett.5b02561

[21] Voisin B., Maurand R., Barraud S., Vinet M., Jehl X., Sanquer M., Renard J., De Franceschi S., Electrical control of *g*-factor in a few hole silicon nanowire MOSFET. Nano Lett. 16, 88–92 (2016). 26599868 10.1021/acs.nanolett.5b02920

[22] Petersson K. D., Smith C. G., Anderson D., Atkinson P., Jones G. A. C., Ritchie D. A., Charge and spin state readout of a double quantum dot coupled to a resonator. Nano Lett. 10, 2789–2793 (2010). 20698590 10.1021/nl100663w

[23] Colless J. I., Mahoney A. C., Hornibrook J. M., Doherty A. C., Lu H., Gossard A. C., Reilly D. J., Dispersive readout of a few-electron double quantum dot with fast rf gate sensors. Phys. Rev. Lett. 110, 046805 (2013). 25166190 10.1103/PhysRevLett.110.046805

[24] Gonzalez-Zalba M. F., Barraud S., Ferguson A. J., Betz A. C., Probing the limits of gate-based charge sensing. Nat. Commun. 6, 6084 (2015). 25600002 10.1038/ncomms7084

[25] House M. G., Kobayashi T., Weber B., Hile S. J., Watson T. F., van der Heijden J., Rogge S., Simmons M. Y., Radio frequency measurements of tunnel couplings and singlet-triplet spin states in Si:P quantum dots. Nat. Commun. 6, 8848 (2015). 26548556 10.1038/ncomms9848 PMC4667619

[26] Johnson A. C., Petta J. R., Taylor J. M., Yacoby A., Lukin M. D., Marcus C. M., Hanson M. P., Gossard A. C., Triplet-singlet spin relaxation via nuclei in a double quantum dot. Nature 435, 925–928 (2005). 15944715 10.1038/nature03815

[27] Voisin B., Nguyen V.-H., Renard J., Jehl X., Barraud S., Triozon F., Vinet M., Duchemin I., Niquet Y.-M., De Franceschi S., Sanquer M., Few-electron edge-state quantum dots in a silicon nanowire field-effect transistor. Nano Lett. 14, 2094–2098 (2014). 24611581 10.1021/nl500299h

[28] Aleksandrov V. N., Gershenzon E. M., Mel’nikov A. P., Rabinovich R. I., Serebryakova N. A., Interaction of A^+^ (D^–^) centers in semiconductors with charged and neutral impurities. JETP Lett. 22, 282–283 (1975).

[29] Koppens F. H. L., Folk J. A., Elzerman J. M., Hanson R., Willems van Beveren L. H., Vink I. T., Tranitz H. P., Wegscheider W., Kouwenhoven L. P., Vandersypen L. M. K., Control and detection of singlet-triplet mixing in a random nuclear field. Science 309, 1346–1350 (2005). 16037418 10.1126/science.1113719

[30] Petta J. R., Johnson A. C., Taylor J. M., Laird E. A., Yacoby A., Lukin M. D., Marcus C. M., Hanson M. P., Gossard A. C., Coherent manipulation of coupled electron spins in semiconductor quantum dots. Science 309, 2180–2184 (2005). 16141370 10.1126/science.1116955

[31] Calvet L. E., Wheeler R. G., Reed M. A., Observation of the linear Stark effect in a single acceptor in Si. Phys. Rev. Lett. 98, 096805 (2007). 17359187 10.1103/PhysRevLett.98.096805

[32] Calvet L. E., Wheeler R. G., Reed M. A., Effect of local strain on single acceptors in Si. Phys. Rev. B 76, 035319 (2007). 10.1103/PhysRevLett.98.096805 17359187

[33] Kartheuser E., Rodriguez S., Group-theoretical study of double acceptors in semiconductors under uniaxial stress. Phys. Rev. B 8, 1556–1570 (1973).

[34] Urdampilleta M., Chatterjee A., Lo C. C., Kobayashi T., Mansir J., Barraud S., Betz A. C., Rogge S., Gonzalez-Zalba M. F., Morton J. J. L., Charge dynamics and spin blockade in a hybrid double quantum dot in silicon. Phys. Rev. X 5, 031024 (2015).

[35] Schroer M. D., Jung M., Petersson K. D., Petta J. R., Radio frequency charge parity meter. Phys. Rev. Lett. 109, 166804 (2012). 23215112 10.1103/PhysRevLett.109.166804

[36] Petersson K. D., McFaul L. W., Schroer M. D., Jung M., Taylor J. M., Houck A. A., Petta J. R., Nature 490, 380–383 (2012). 23075988 10.1038/nature11559

[37] Bir G. L., Butikov E. I., Pikus G. E., Spin and combined resonance on acceptor centres in Ge and Si type crystals—II: The effect of the electrical field and relaxation time. J. Phys. Chem. Solids 24, 1475–1486 (1963).

[38] Srinivasa V., Nowack K. C., Shafiei M., Vandersypen L. M. K., Taylor J. M., Simultaneous spin-charge relaxation in double quantum dots. Phys. Rev. Lett. 110, 196803 (2013). 23705734 10.1103/PhysRevLett.110.196803

[39] Yang C. H., Rossi A., Ruskov R., Lai N. S., Mohiyaddin F. A., Lee S., Tahan C., Klimeck G., Morello A., Dzurak A. S., Spin-valley lifetimes in a silicon quantum dot with tunable valley splitting. Nat. Commun. 4, 2069 (2013). 23804134 10.1038/ncomms3069

[40] Bulaev D. V., Loss D., Spin relaxation and decoherence of holes in quantum dots. Phys. Rev. Lett. 95, 076805 (2005). 16196813 10.1103/PhysRevLett.95.076805

[41] Stano P., Fabian J., Orbital and spin relaxation in single and coupled quantum dots. Phys. Rev. B 74, 045320 (2006). 10.1103/PhysRevLett.96.186602 16712384

[42] Bir G. I., Butikov E. I., Pikus G. E., Spin and combined resonance on acceptor centres in Ge and Si type crystals—I: Paramagnetic resonance in strained and unstrained crystals. J. Phys. Chem. Solids 24, 1467–1474 (1963).

[43] R. Winkler, *Spin-orbit Coupling Effects in Two-Dimensional Electron and Hole Systems* (Springer-Verlag, Berlin Heidelberg, 2003).

[44] Köpf A., Lassmann K., Linear Stark and nonlinear Zeeman coupling to the ground state of effective mass acceptors in silicon. Phys. Rev. Lett. 69, 1580–1583 (1992). 10046258 10.1103/PhysRevLett.69.1580

[45] Stegner A. R., Tezuka H., Andlauer T., Stutzmann M., Thewalt M. L. W., Brandt M. S., Itoh K. M., Isotope effect on electron paramagnetic resonance of boron acceptors in silicon. Phys. Rev. B 82, 115213 (2010).

[46] Koppens F. H. L., Buizert C., Tielrooij K. J., Vink I. T., Nowack K. C., Meunier T., Kouwenhoven L. P., Vandersypen L. M. K., Driven coherent oscillations of a single electron spin in a quantum dot. Nature 442, 766–771 (2006). 16915280 10.1038/nature05065

[47] Hile S. J., House M. G., Peretz E., Verduijn J., Widmann D., Kobayashi T., Rogge S., Simmons M. Y., Radio frequency reflectometry and charge sensing of a precision placed donor in silicon. Appl. Phys. Lett. 107, 093504 (2015).

[48] Schechter D., Theory of shallow acceptor states in Si and Ge. J. Phys. Chem. Solids 23, 237–247 (1962).

[49] Ramdas A. K., Rodriguez S., Spectroscopy of the solid-state analogues of the hydrogen atom: Donors and acceptors in semiconductors. Rep. Prog. Phys. 44, 1297 (1981).

[50] Smit G. D. J., Rogge S., Caro J., Klapwijk T. M., Group-theoretical analysis of double acceptors in a magnetic field: Identification of the Si:B^+^ ground state. Phys. Rev. B 69, 085211 (2004).

[51] Bhattacharjee A. K., Rodriguez S., Group-theoretical study of the Zeeman effect of acceptors in silicon and germanium. Phys. Rev. B 6, 3836 (1972).

