# Supplementary material for: Readout and control of the spin-orbit states of two coupled acceptor atoms in a silicon transistor
Source: Sci Adv. 2018 Dec 7;4(12):eaat9199. doi: 10.1126/sciadv.aat9199 (PMC6286166; doi:10.1126/sciadv.aat9199)
Supplement: http://advances.sciencemag.org/cgi/content/full/4/12/eaat9199/DC1 [file supp_4_12_eaat9199__index.html]

Science Advances | Science Advances

## Supplementary Materials

**This PDF file includes:**

- Section S1. Estimation of tunnel rates
- Section S2. Acceptor-based single-atom qubits
- Section S3. Two-hole states
- Section S4. Relaxation mechanisms
- Section S5. (1,1)→(2,0) transition energies
- Fig. S1. Transport spectroscopy images.
- Fig. S2. Frequency domain analysis of the reflectometry signal.
- Fig. S3. Interacceptor continuous-wave microwave excitation experiment.
- Fig. S4. Examples of the Zeeman effect on *A*0 states.
- Fig. S5. Examples of the Zeeman effect on two-hole states.
- Fig. S6. Relaxation rate models.
- References (*47*–*51*)

Download PDF

**Files in this Data Supplement:**

- Adobe PDF - aat9199\_SM.pdf
