# Supplementary material for: Readout and control of the spin-orbit states of two coupled acceptor atoms in a silicon transistor
Source: Sci Adv. 2018 Dec 7;4(12):eaat9199. doi: 10.1126/sciadv.aat9199 (PMC6286166; doi:10.1126/sciadv.aat9199)
Supplement: http://advances.sciencemag.org/cgi/content/full/4/12/eaat9199/DC1 [file aat9199_SM.pdf]

Science Advances
AAAS

advances.sciencemag.org/cgi/content/full/4/12/eaat9199/DC1

Supplementary Materials for

Readout and control of the spin-orbit states of two coupled acceptor atoms in a silicon transistor

Joost van der Heijden, Takashi Kobayashi, Matthew G. House, Joe Salfi, Sylvain Barraud, Romain Laviéville, Michelle Y. Simmons, Sven Rogge*

*Corresponding author. Email: s.rogge@unsw.edu.au

Published 7 December 2018, *Sci. Adv.* **4**, eaat9199 (2018)
DOI: 10.1126/sciadv.aat9199

This PDF file includes:

Section S1. Estimation of tunnel rates
Section S2. Acceptor-based single-atom qubits
Section S3. Two-hole states
Section S4. Relaxation mechanisms
Section S5. (1,1)→(2,0) transition energies
Fig. S1. Transport spectroscopy images.
Fig. S2. Frequency domain analysis of the reflectometry signal.
Fig. S3. Interacceptor continuous-wave microwave excitation experiment.
Fig. S4. Examples of the Zeeman effect on A^0 states.
Fig. S5. Examples of the Zeeman effect on two-hole states.
Fig. S6. Relaxation rate models.
References (*47–51*)

Supplementary materials

Section S1. Estimation of tunnel rates

In the RF gate reflectometry experiments shown in the main text, a driving frequency of 583 MHz is used, which is close to the resonant frequency and ideal to distinguish the acceptor-lead and inter-acceptor transitions. The full frequency response of the reflected signal from the LC-circuit reveals more information about the tunnel rates involved in the experiment (*47*).

Frequency domain measurements of the amplitude and phase response around the resonance of the LC circuit are shown in figs. S2A and S2B. These measurements show that the acceptor-lead signal mainly changes the amplitude of the reflected signal, while the inter-acceptor signal mainly reduces the resonant frequency. These changes to the resonant behavior as a function of the drive frequency are made more clear by plotting the difference in amplitude and phase response compared to the response when no charge transitions occur (the off-resonance response). In figs. S2C and S2D these differences are shown as a function of the drain voltage near the two-hole transitions, specifically on the cut-through of the transition lines in Fig. 2A at a backgate voltage of 1.93V. The different responses of the acceptor-lead and inter-acceptor tunnel processes are highlighted by showing the line cuts from figs. S2C and S2D for these different transitions in figs. S2E and S2F.

This difference in response of the LC-circuit can be explained by a difference in tunnel rate between these two processes (*25*). When the tunnel rate is much faster than the resonant frequency ($\Gamma >> \omega$), the tunnel process will predominantly add capacitance to the circuit (dispersive response), which in turn shifts the resonance to a lower frequency. This is observed for the inter-acceptor tunneling process, thereby revealing a tunnel rate much faster than the resonant frequency. When the tunnel rate is of the order of the resonant frequency ($\Gamma \sim \omega$), the tunnel process will predominantly add resistance to the circuit (dissipative response). This added resistance can either bring the circuit closer or further away from its ideal matching condition, depending on the initial impedance of the circuit, thereby either reducing or increasing the reflection amplitude. A reduction in reflection amplitude is observed for the acceptor-lead tunneling process, thereby revealing a tunnel rate of the order of the resonant frequency.

To find the tunnel coupling between the two acceptor atoms, we probe the resonance between the bonding and anti-bonding singlet *S* states, as shown in the inset of fig. S3, in accordance with a measurement on two electrons on a hybrid donor-quantum dot system (*34*). Here we make use of the bias-tee coupled to the drain lead, as shown in Fig. 1A in the main text. The inter-acceptor signal is probed with RF gate reflectometry, while continuous wave microwaves are applied to the drain electrode. When the transition between the bonding and anti-bonding *S* states is excited by the applied microwaves, the occupation of the bonding state is lowered, while the occupation of the anti-bonding state is increased. As the bonding and anti-bonding singlet states give rise to an opposite response of the resonator (*34*), the observed $\Delta\phi_{refl}$ is expected to decrease when the occupation in the anti-bonding state is increased and completely disappear when the occupations in the system become unpolarised.

In order to keep the input power of the applied microwaves constant during the experiment, the splitting of the acceptor-drain signal is also probed during the experiment. The splitting of this signal has no dependence on the microwave frequency and is solely dependent on the microwave power applied at the drain. Using this measurement we can calibrate for any frequency dependent losses in the system and use the same input power for every frequency. The response of the inter-acceptor tunnel signal is shown in fig. S3 as $\Delta\phi_{refl}/\Delta\phi_0$, with $\Delta\phi_0$ being the signal without microwaves. The resonance dips at a frequency of the 8.6 ± 0.6 GHz, which demonstrates a tunnel coupling between the atoms of 4.3 ± 0.3 GHz. The width of this resonance indicates a T_2^* for these charge states of ~ 200 ps, similar to what was found for a two-electron system on a hybrid donor-quantum dot (*34*).

An estimate for the tunnel rate to the second acceptor, although not visible in the reflectometry measurements, can be found by studying the transport data. In fig. S1B the measured current through the double acceptor system at a drain voltage of 3 mV is shown. The tunnel current within the bias triangles is not constant, but decreases closer to the resonant tunneling line of the second acceptor. This points to a stronger off-resonant tunnel rate. The maximum measured current is ~ 1 pA, which relates to a total tunnel rate of ~ 6 MHz. As the other tunnel rates involved in this charge transfer have already been established to be equal to or faster than the tank circuit resonance frequency of 583 MHz, this can be used as a direct estimation for the off-resonant tunnel rate for the second acceptor and as an upper bound for the resonant tunnel rate.

Section S2. Acceptor-based single-atom qubits

In the last decade, a lot of progress has been made in achieving quantum control of single phosphorous donors in silicon (*1, 2)*. The minimal spin-orbit interaction in the conduction band of silicon and the consequent little coupling to electric noise sources is one of the main reasons for the remarkably long coherence times achieved. With the possibility to minimize the magnetic noise through isotope purification to ^{28}Si, very long coherence times and high fidelity quantum gates have been demonstrated for both the electron and nuclear spin of phosphorous atoms (*2*). Acceptor atoms have received considerably less attention. In contrast to the conduction band, a strong spin-orbit interaction is present for holes in the valence band of silicon. Although this will inevitably cause shorter coherence times compared to donor atoms, it will also allow for fast electrical spin manipulation and long distance qubit coupling via microwave cavities.

The valence band of silicon is p-type (l=1), where the two bands at the top of this band carry a total angular momentum of j=3/2, with the heavy hole ($m_j = \pm 3/2$) and the light hole ($m_j = \pm 1/2$) bands degenerate at the Γ-point. These bands are separated from the j=1/2 split-off band due to the spin-orbit interaction by 44 meV. The single hole states of an acceptor are build from the different valence bands, and in bulk silicon the degeneracy between heavy and light holes is preserved in the acceptor ground state, as the spherical symmetric confinement of the atom does not break the cubic silicon symmetry. The ground state of a neutral boron atom has a fourfold degenerate Γ_8 symmetry and a binding energy of 46 meV and the full energy spectra of acceptors in silicon are well understood (*48, 49*).

The effect of strain, electric field and magnetic field on these states is well described by Bir et al. (*37, 42*). Strain and electric field split the heavy and light hole manifolds, leading to a splitting of Δ_{LH} at zero magnetic field. A magnetic field splits all four spin-states, where this Zeeman effect strongly depends on the local environment of the atom (see fig. S4). The key properties of acceptor-based spin-orbit qubits, such as the dipole coupling and decoherence time between the chosen qubit states, are predicted to strongly depend on these changes to the symmetry of the silicon crystal and the confinement of the hole (*10-12, 37, 42*). An optimal regime, where the coherence and relaxation times are prolonged while the strong electric dipole coupling is maintained, has been predicted in the small Δ_{LH} splitting regime, with a strong mixing between the heavy and light hole states (11).

Section S3. Two-hole states

The two atoms probed in the experiments presented in the main text are found to have heavy hole ground states. The Δ_{LH} is assumed to be different for the two acceptors, as they experience a different local environment. As an example, the Zeeman effect is calculated for two different local environments where the heavy holes form the ground state at zero magnetic field, shown in fig. S4. For the first example we consider the situation where a compressive strain of 1.2×10^{-4} is present parallel to the magnetic field and an electric field of 0.6 MV/m is present perpendicular to the magnetic field, see fig. S4A. In fig. S4B the situation with half of the strain and double the electric field is considered. In this second example, we note that if a linear approximation is taken for the hole-spin states in the high magnetic field region and traced back to zero magnetic field (dashed lines), a different heavy-light hole splitting is found than at zero magnetic field. This effect has been observed in our experiment presented in Fig. 4 of the main text.

For the doubly occupied and positively charged acceptor state (A^+) the exchange energy between two holes from the same manifold far exceeds the heavy-light hole splitting (J_{HH}, $J_{LL} >> \Delta_{LH}$). Due to this exchange interaction, only 6 out of the 16 possible two-hole states can be bound to a boron atom in silicon (*50*). A similar sixfold manifold is found for two-holes bound to two closely placed boron atoms (*15*) and the neutral states of two holes bound to group II acceptors (*33, 51*). As these six states are build from combinations of different spin states, one heavy hole singlet state, one light hole singlet state and four light/heavy-quadruplet states are found, which we will write as

$$|S_{HH}\rangle = 1/\sqrt{2}\left(|\Uparrow\Downarrow\rangle - |\Downarrow\Uparrow\rangle\right)$$

$$|S_{LL}\rangle = 1/\sqrt{2}\left(|\uparrow\downarrow\rangle - |\downarrow\uparrow\rangle\right)$$

$$|Q_{LH}^{2-}\rangle = 1/\sqrt{2}\left(|\Downarrow\downarrow\rangle - |\downarrow\Downarrow\rangle\right)$$

$$|Q_{LH}^{-}\rangle = 1/\sqrt{2}\left(|\Downarrow\uparrow\rangle - |\uparrow\Downarrow\rangle\right)$$

$$|Q_{LH}^{+}\rangle = 1/\sqrt{2}\left(|\Uparrow\downarrow\rangle - |\downarrow\Uparrow\rangle\right)$$

$$|Q_{LH}^{2+}\rangle = 1/\sqrt{2}\left(|\Uparrow\uparrow\rangle - |\uparrow\Uparrow\rangle\right)$$

where $\Uparrow,\Downarrow$ represent the heavy hole states and $\uparrow,\downarrow$ represent the light hole states. Furthermore, when an exchange interaction between the heavy and light holes is introduced, J_{LH}, the Q_{LH} state splits into a singlet and triplet state (*11*). In fig. S5A an example of the magnetic field dependence of the A^+-states of an acceptor is shown, using the A^0 states from fig. S4B and neglecting any exchange between the light and heavy holes ($J_{LH} = 0$). The colors in the figure show the heavy-hole (red) and light-hole (blue) character of these states, where the states built from one heavy and one light hole turn purple. The splitting between the heavy hole singlet and the light hole singlet (E_{SLL} - $E_{SHH} = 2\Delta_{LH}$) is found to be different in the high magnetic field limit compared to the zero magnetic field splitting, in line with was found in fig. S4B.

In the situation where each hole is bound to a different acceptor, the two hole state can be described as a product state of the single particle states, leading to an energy spectrum of 16 states divided into 4 fourfold degenerate manifolds at zero magnetic field. Each fourfold manifold splits into a singlet and a triplet state when an exchange interaction is introduced. In our experiments the heavy-hole manifold has the lowest energy at zero magnetic field and can be described as

$$|S_{HH}\rangle = 1/\sqrt{2}\left(|\Uparrow,\Downarrow\rangle - |\Downarrow,\Uparrow\rangle\right)$$

$$|T_{HH}^{-}\rangle = |\Downarrow,\Downarrow\rangle$$

$$|T_{HH}^{0}\rangle = 1/\sqrt{2}\left(|\Uparrow,\Downarrow\rangle + |\Downarrow,\Uparrow\rangle\right)$$

$$|T_{HH}^{+}\rangle = |\Uparrow,\Uparrow\rangle$$

The energy splitting to the next two manifolds is given by the Δ_{LH} of each acceptor, where these two-hole states consist of one heavy and one light hole state. Finally, the light-hole manifold is split from the heavy-hole manifold by the sum of both Δ_{LH}'s. In fig. S5B an example of such a (1,1) level configuration is shown for a combination of the A^0-states presented in figs. S4A and S4B. Although many level crossings are observed, we highlight the fact that in this situation where the heavy holes form the ground state manifold, the ground state in the (1,1) region remains the *T* state for any positive magnetic field.

Section S4. Relaxation mechanisms

At magnetic fields much higher than the $B_{S\rightarrow Q}$ mixing point the dominant relaxation mechanism comes from either the relaxation to the (1,1) *T* state or the relaxation to the (2,0) *Q* state, given by Eq. 1 in the main text. In fig. S6A the fits of the relaxation rate data with a combination of Eqs. 1 and 2 is given for the relaxation process to the *T* state (blue) and the *Q* state (red) are shown. Our data is insufficient to determine if a $(\hbar\omega)^3$ or $(\hbar\omega)^5$ relaxation mechanism is dominant at high magnetic fields. The relaxation amplitudes extracted from these two fits, which are interpreted as the relaxation time at a field of 1 T relative to the corresponding level-crossing, is found to be 0.5 μs for A_{SQ} and 5 μs for A_{ST}. Both of these values are in good agreement with the theoretical predictions for light-heavy hole relaxation (T_1 = 0.14 μs at 1T (*9*)) and heavy-heavy hole relaxation (T_1 = 40 μs at 0.5T (*10*)). Figure S6B shows the extracted relaxation rates on a larger scale of $1/T_1$, demonstrating the more than 3 orders of magnitude increase in relaxation rate at the hotspot. Furthermore, it reveals that if this hotspot behavior can be moved to a higher magnetic field, by increasing the heavy-light hole splitting or decreasing the g-factor, relaxation times longer than 1 ms could be achieved at magnetic fields around 0.5T.

Section S5. (1,1)→(2,0) transition energies

In the transport measurements shown in Fig. 4 in the main text the (1,1) → (2,0) transition energies at zero magnetic field and as a function of magnetic fields up to 3 T are probed. In these experiments, a voltage of +10 mV is applied to the drain, while the current is measured at the source. This bias direction gives a transport direction for the holes from the drain to the source. From the RF gate reflectometry experiments, as shown in Fig. 2 in the main text, it is deduced that the acceptor with a charge transition from 0 to 1 hole has a stronger tunnel coupling to the drain (detectable with RF reflectometry) than the acceptor with a charge transition from 1 to 2 holes (not detectable with RF reflectometry). It follows that the probed sequential tunnelling involves the (1,1) → (2,0) transition, where the two observed bias triangles are formed by the two following hole charge transfer processes

$$(1,0)\rightarrow(1,1)\rightarrow(2,0)\rightarrow(1,0)$$

$$(2,1)\rightarrow(1,1)\rightarrow(2,0)\rightarrow(2,1)$$

The current measured within the bias triangle region is around 5 pA at the baseline at zero magnetic field and declining into the measurement noise floor of ~ 100 fA at the other side of the triangle, indicating a total tunnel rate of the sequential tunnel process around 30 MHz and lower. We recall that the resonant tunnel-rate between the (1,1) and (2,0) *S* states was measured at 4.3 GHz and the tunnel-rate from the drain to the acceptor was found to be around the resonant frequency of 583 MHz, indicating that the tunnel-rate between the second acceptor and the source is the limiting tunnel process. However, we note that due to off-resonant tunneling or tunneling between two states consisting of different spins, the inter-acceptor tunnel rate can be strongly suppressed, thereby possibly becoming the limiting tunnel process. Furthermore, the ratio between this inter-acceptor tunnel rate and the relaxation rate of each excited state in the (1,1)-configuration determines if this excited state will have any effect on the measured current. The transport measurement presented in the main text can be explained by only taking the heavy-hole manifold of the (1,1)-configuration into account. This indicates that any state which includes any light-hole has a fast relaxation to the heavy hole ground state, which matches with the fast light-to-heavy hole relaxation process observed as a relaxation hotspot in the main text.

SUPPLEMENTARY FIGURES

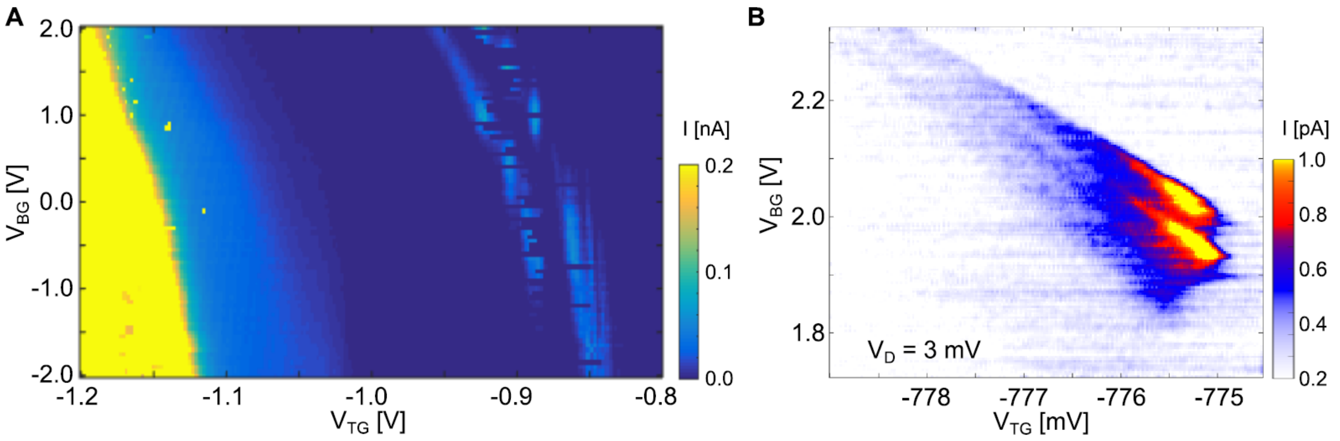

Fig. S1. Transport spectroscopy images. (**A**) Large scale current map as a function of top gate and back gate voltages, which shows a back gate dependent turn-on of the transistor around a top gate voltage of -1.1 V. The charging and discharging of charge traps in the channel, activated at these lower top gate voltages, cause jumps in the current. (**B**) Current I as a function of the top and back gate voltages at a 3 mV V_D. The bias triangles show a maximum current of ~1 pA.

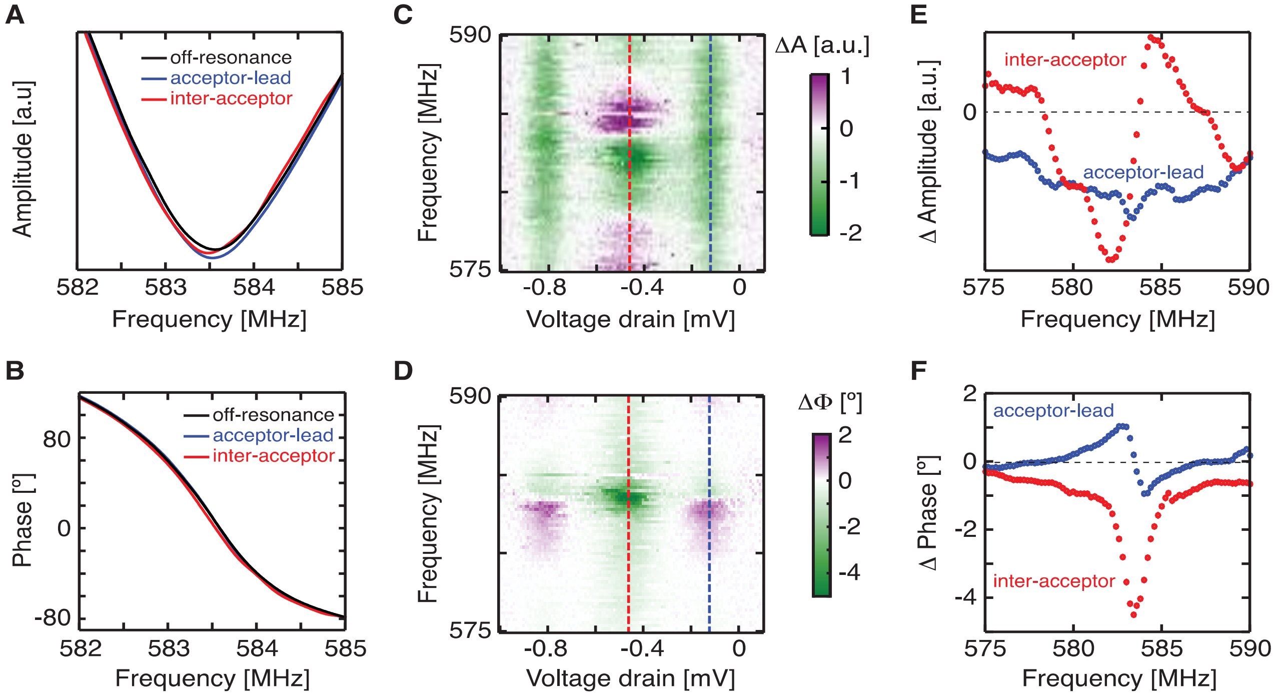

Fig. S2. Frequency domain analysis of the reflectometry signal. (**A**), [(**B**)] Amplitude [phase] response of the tank circuit as a function of frequency. (**C**), [(**D**)] Amplitude [phase] shift relative to the off-resonant signal as a function of frequency on a line cut through the (1,1)-(2,0) transition, with V_{BG} = 1.93 V and V_{TG} = -777 mV. (**E**), [(**F**)] Amplitude [phase] shift for the line cuts on the red (inter-acceptor signal) and blue (acceptor-lead signal) dotted lines in (C) [(D)].

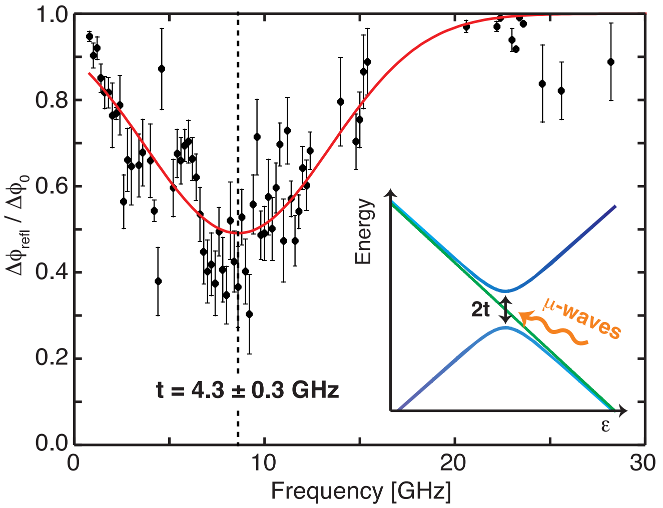

Fig. S3. Interacceptor continuous-wave microwave excitation experiment. The change in phase shift of the inter-acceptor tunnel signal as a function of microwave frequency, using a constant microwave power applied to the drain electrode of the transistor. The resonant dip reveals the tunnel coupling between the atoms measured as $t = 4.3 \pm 0.3$ GHz. The inset shows a schematic representation depicting the excitation from the bonding to the anti-bonding singlet state with microwaves.

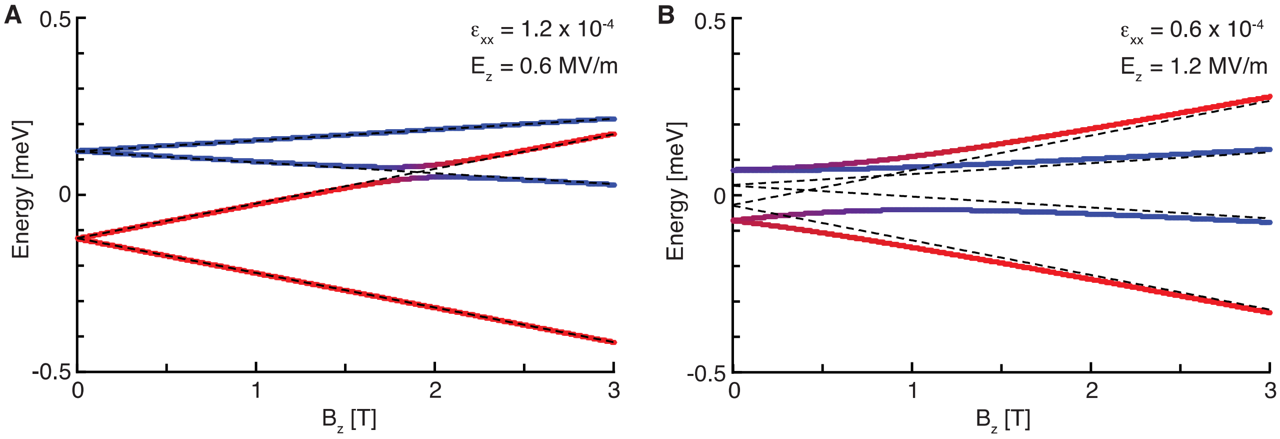

Fig. S4. Examples of the Zeeman effect on A^0 states. (**A**) Magnetic field dependence of the energy levels of a hole bound to a single boron acceptor in silicon with a compressive strain of 1.2×10^{-4} parallel with the magnetic field and an electric field of 0.6 MV/m perpendicular to the magnetic field. (**B**) Magnetic field dependence of the energy levels of a hole bound to a single boron acceptor in silicon with a compressive strain of 0.6×10^{-4} parallel with the magnetic field and an electric field of 1.2 MV/m perpendicular to the magnetic field. Dashed lines show the linear projection of the high magnetic field Zeeman effect, showing a change in Δ_{LH} in (B).

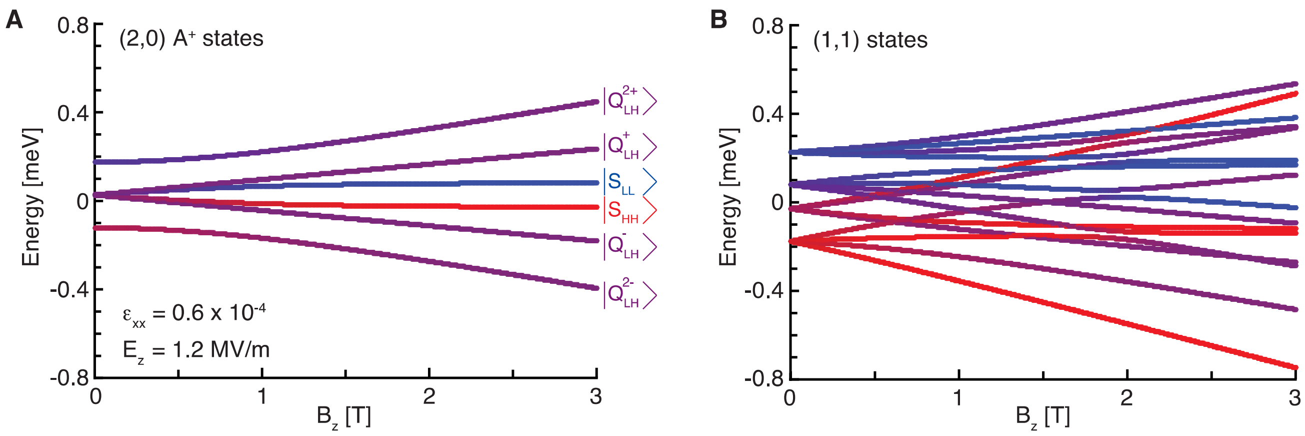

Fig. S5. Examples of the Zeeman effect on two-hole states. The light or heavy hole character of each state is represented by the line color, where red represents heavy hole character and blue represents light hole character. (**A**) A^+ energies for the same local environment as in fig. S4B and $J_{LH} = 0$. A clear difference in splitting between the S_{HH} and S_{LL} states in the low and high magnetic field limit is visible. (**B**) The (1,1)-configuration product states of the two single hole states described in figs. S3A and S3B.

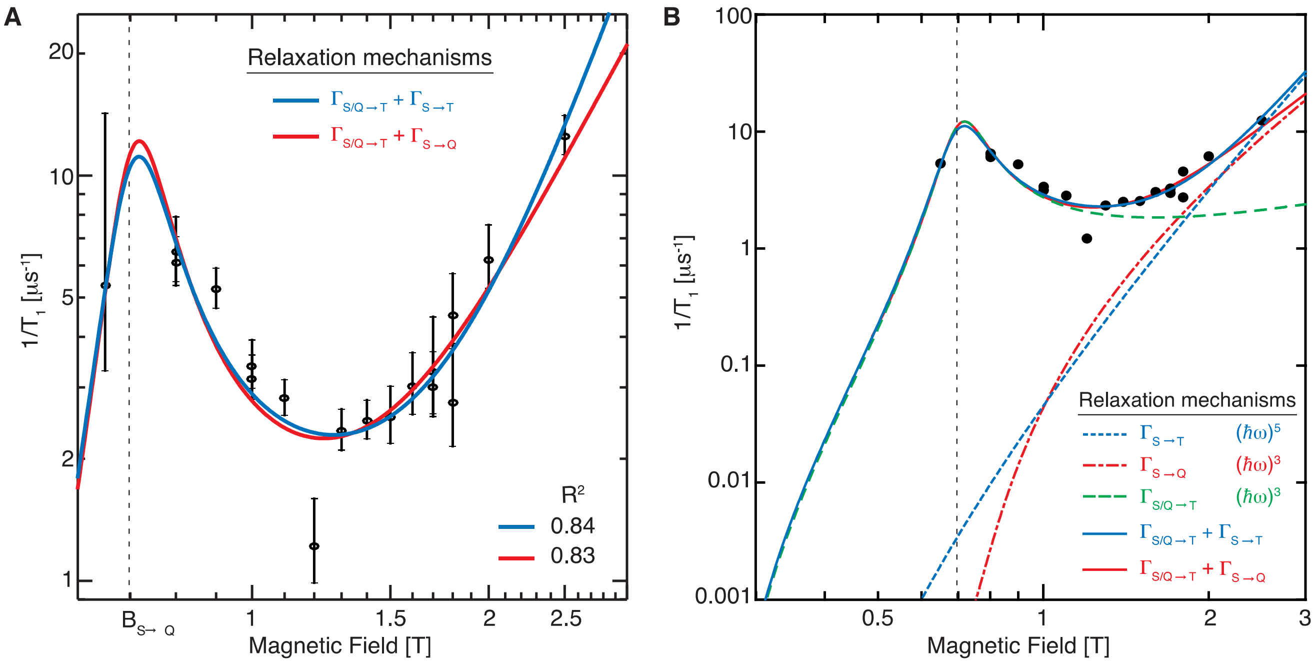

Fig. S6. Relaxation rate models. (**A**) Comparison between the fit of the relaxation rate data with a model combining the hotspot-relaxation mechanism with either the *S* to *T* relaxation (blue) or with the *S* to *Q* relaxation (red). Both fits have a comparable value for R^2. (**B**) Comparison of the modeled relaxation mechanisms on a larger scale of magnetic field and T_1^{-1}.
